# The Role of Extracellular Matrix in Skin Wound Healing

**DOI:** 10.3390/jcm10245947

**Published:** 2021-12-18

**Authors:** Nikolai N. Potekaev, Olga B. Borzykh, German V. Medvedev, Denis V. Pushkin, Marina M. Petrova, Artem V. Petrov, Diana V. Dmitrenko, Elena I. Karpova, Olga M. Demina, Natalia A. Shnayder

**Affiliations:** 1 Department of Skin Disease and Cosmetology, Pirogov Russian National Research Medical University, 117997 Moscow, Russia; klinderma@mail.ru (N.N.P.); elena-karpova@inbox.ru (E.I.K.); demina.om@mail.ru (O.M.D.); 2 Shared Core Facilities “Molecular and Cell Technologies”, V. F. Voino-Yasenetsky Krasnoyarsk State Medical University, 660022 Krasnoyarsk, Russia; stk99@yandex.ru (M.M.P.); artvpetrov@mail.ru (A.V.P.); mart2802@yandex.com (D.V.D.); 3 Department of Hand Surgery with Microsurgical Equipment, R. R. Vreden National Medical Research Centre for Traumatology and Orthopedics, 195427 Saint Petersburg, Russia; dr.medvedev.g@yandex.ru; 4 Medical Faculty, Saint Petersburg State University, 199034 Saint Petersburg, Russia; pushkindv00@gmail.com; 5 Institute of Personalized Psychiatry and Neurology, Shared Core Facilities, V. M. Bekhterev National Medical Research Centre for Psychiatry and Neurology, 192019 Saint Petersburg, Russia

**Keywords:** wound healing, extracellular skin matrix, violation of regeneration

## Abstract

Impaired wound healing is one of the unsolved problems of modern medicine, affecting patients’ quality of life and causing serious economic losses. Impaired wound healing can manifest itself in the form of chronic skin wounds or hypertrophic scars. Research on the biology and physiology of skin wound healing disorders is actively continuing, but, unfortunately, a single understanding has not been developed. The attention of clinicians to the biological and physiological aspects of wound healing in the skin is necessary for the search for new and effective methods of prevention and treatment of its consequences. In addition, it is important to update knowledge about genetic and non-genetic factors predisposing to impaired wound healing in order to identify risk levels and develop personalized strategies for managing such patients. Wound healing is a very complex process involving several overlapping stages and involving many factors. This thematic review focuses on the extracellular matrix of the skin, in particular its role in wound healing. The authors analyzed the results of fundamental research in recent years, finding promising potential for their transition into real clinical practice.

## 1. Introduction

The ability of tissues to recover after damage is one of the fundamental properties of all organisms that underlies the maintenance of the body’s homeostasis [1]. The skin is the most important barrier of the body against the damaging effects of external factors; therefore, to maintain the integrity and homeostasis of the skin, there are complex mechanisms for self-defense and restoration of the integrity of the skin after damage. The skin is a more accessible organ than others for experiments in animals and humans; therefore, most of the fundamental research in the field of regenerative biology aimed at studying the mechanisms of repair is carried out on the skin, with the subsequent comparison of the results obtained with the mechanisms of regeneration in other epithelial–mesenchymal organs. All living organisms have mechanisms for tissue restoration after tissue damage, while in most vertebrates in the postembryonic period, healing occurs through scar formation [2]. Only fetal wounds can fully restore the integrity, biomechanics, and function of tissues, with the best regenerative wound healing. Wounds in adults are not capable of complete regeneration, and the wound surface is replaced by scar tissue. With the formation of scar tissue, fibrillar collagen accumulates, with a prolonged inflammatory process or impaired neovascularization. This scar tissue is less tear-resistant. At the same time, whereas the usual arrangement of fibrillar collagen fibers in the skin is in the form of a network, in scar tissue, fibrillar collagen accumulates in large quantities in the form of thick bundles running parallel to the length of the tissue.

Molecular and cellular causes of poor wound healing on the skin are being actively studied, but presently, the issue of prevention and effective treatment for wound healing remains open. Two questions are being actively discussed: “Why do some people experience pathological wound healing even with relatively minor trauma? How to prevent the formation of unfavorable scars on the skin?” [3].

Poor wound healing is a consequence of the disturbance of the action of factors in the process of tissue repair in response to damage and is the cause of lengthened processes of wound healing after surgery and skin damage, the formation of hypertrophic or atrophic scars, chronic trophic ulcers, and other pathological conditions [4]. Therefore, wounds that fail to proceed through the normal phases of wound healing are defined as chronic wounds [5]. The failure of cellular processes during the tissue repair process can be associated with the clinical state of the body (vascular diseases, diabetes mellitus, aging) and can also be caused by genetic variations in genes responsible for the components involved in the tissue repair process. In the latter case, research is focused on a genetic predisposition to pathological scarring of wounds [3], which refers to a multifactorial pathology [6,7]. Genetic predisposition is currently the least-studied predictor of impaired wound healing. There is clinical interest in the study of candidate genes in combination with the assessment of other factors, which will make it possible to assess the cumulative risk of impaired wound healing in patients undergoing surgical and nonsurgical treatments for skin lesions, including patients with atrophic scars. Cumulative assessment of the risk of damage to skin wounds is an important aspect of the development of a personalized algorithm for the management of such patients [8].

This thematic review focuses on the extracellular matrix of the skin, its involvement in wound healing, and genetic predictors of impaired wound healing. The aim of this review was to integrate data on previously studied candidate genes and information on the participation of the extracellular matrix at different stages of wound healing. This approach allowed us to take a fresh look at the problem of wound healing.

## 2. Materials and Methods

This review was carried out over June and July 2021 across 4 databases: PubMed, Scopus, Web of Science, and Ciberleninka (Russian) (2010–2021). The following combination of subject words and keywords was used: wound, injuries, healing, regeneration, scar, extracellular matrix. The logical relationship was created with “OR” and “AND”, and the search formula was thereafter developed according to the different databases. With regard to repeatedly reported articles, only the latest or most comprehensive one was included.

## 3. Results

Four hundred fifty-four articles were selected by the preliminary screening. Only 93 studies and reviews were kept after screening titles, abstracts, and full texts.

### 3.1. Stages of Wound Healing

**Figure 1:**
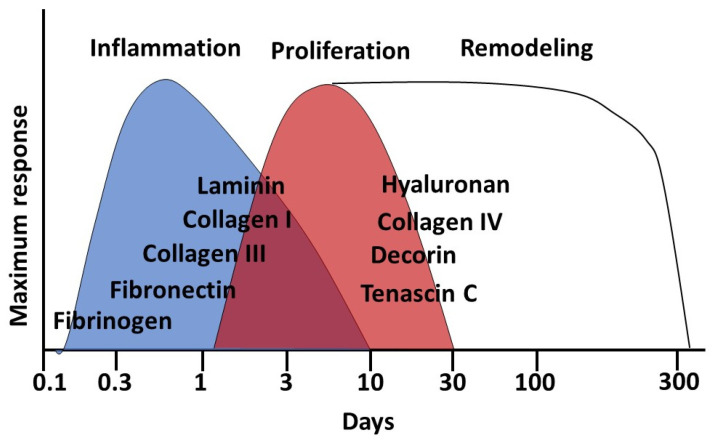
Wound healing stages and main involved components of the extracellular matrix of the skin.

Wound healing in the skin occurs in three overlapping stages, which determine the further process [9,10] (Figure 1):

- inflammation (with hemostasis);
- proliferation;
- remodeling.

The repair response to restore tissue integrity and homeostasis occurs within a few seconds; the cellular components of the immune system (neutrophils, monocytes, lymphocytes, and dendritic cells) are activated. Then, there is a cascade of the blood coagulation system and, finally, the activation of the inflammatory pathway.

When the skin is damaged, the coagulation cascade is immediately activated, and a fibrin clot is formed, which provides hemostasis and the basic architecture of the matrix for the migration of other cells. Platelets trapped in the clot release cytokines, hormones, and chemokines. Among the growth factors, we can highlight platelet-derived growth factor (PDGF), transforming growth factor-β (TGF-β), epidermal growth factor (EGF), and fibroblast growth factor (FGF). These properties are reflected in the clinical use of platelet-rich plasma to promote wound healing [11].

At the stage of an early inflammatory response, local and systemic defense responses are mobilized. The stage of inflammation in chronic wounds is prolonged, an imbalance occurs between inflammatory and anti-inflammatory signals, and chronic inflammation develops, which does not progress and leads to an imbalance in the environment, disrupting the wound healing processes [12].

Pro-inflammatory cell infiltration, consisting of neutrophils and macrophages, slows down wound healing [13]. With a local increase in the level of pro-inflammatory cytokines—interleukin 1 beta (IL-1β) and tumor necrosis factor alpha (TNF-α)—the inflammation phase is prolonged; wound healing is delayed; the activity of matrix metalloproteinases (MMPs), which destroy the extracellular matrix, increases; and migration worsens cells [14]. In chronic wounds, the levels of MMPs (especially collagenase and gelatinase) and serine proteases, which are capable of destroying components of the extracellular matrix, are significantly increased. Further, the levels of key growth factors necessary for skin remodeling in the wound area are increased. Inflammatory cells perform many tasks in a wound: wound care, synthesis of chemokines, and producing growth factors. If the coordinated process of inflammatory cells is disturbed, a chronic wound and the progression of fibrosis in the wound can form [15].

Inflammation is a key phase of wound healing, and the amount of scar tissue after wound healing depends on the characteristics of the inflammatory response [16]. Prolonged acute inflammation can slow down early tissue repair and fibrotic processes, thereby impairing the proliferation of keratinocytes and the recruitment of profibrotic macrophages.

Neutrophils at an early stage of inflammation kill microorganisms that are trapped after violation of the skin barrier [17]. Macrophages at an early stage purify neutrophils after apoptosis of damaged skin cells and produce signals that cause subsequent scar formation [18,19]. Then, they change phenotype and participate in the cell proliferation reaction, producing anti-inflammatory cytokines and extracellular matrix [20]. The role of mast cells is not fully understood. However, the hypothesis is that even with depletion of mast cells, normal wound healing can occur [21,22]. An alternative hypothesis is that a local increase in the level of mast cells during the development of hypertrophic scars and fibrosis plays an important role in wound healing [23,24].

After restoring tissue homeostasis, the inflammatory process must be controlled and turned off to prevent the development of a pathological process. The result of the stage of inflammation, which lasts an average of 48 h and is the cleansing of the damaged area of the skin. Then, the stage of proliferation (epithelization) is started; it overlaps with the inflammation stage and usually lasts up to 2–3 weeks after injury [25]. At the stage of proliferation, re-epithelialization occurs due to proliferation of keratinocytes at the edge of the wound; induction of angiogenesis; and proliferation and production of granulation tissue by fibroblasts, consisting of procollagen, elastin, and hyaluronic acid. As a result of mechanical stress and TGFβ1 signaling, many cells migrate into the wound: fibroblasts; pericytes; adipocytes; resident mesenchymal progenitor cells; and mesenchymal stem cells.

Moreover, some fibroblasts under the action of TGF-β differentiate into myofibroblasts, which are also capable of multiplying and secreting extracellular matrix proteins such as collagens I and III and fibronectin [26]. Myofibroblasts have a contractile function. They attach to the extracellular matrix via integrins and contract via alpha smooth muscle actin (αSMA). These rich stress fibers reduce wound size by promoting wound closure in the skin [27].

At this stage, the reticular layer of the dermis is most sensitive to external influences. In this regard, with the additional influence of external unfavorable factors, the scarring of the wound is disturbed, and if the coordinated work of all components involved in wound healing is disrupted, persistent inflammation occurs, leading to the formation of hypertrophic or keloid scars in the skin [28].

The final stage of wound healing is the recovery stage (or remodeling stage). This stage can last for up to a year after skin injury (up to 24 months after skin burns). Skin remodeling begins immediately after the closure of the skin wound. At the same time that the wound shrinks, collagen remodeling and restructuring of the entire unorganized extracellular matrix occurs in it [29]. At this stage, there is a decrease in vascularization. Initially, cells involved in the previous stages (macrophages, endothelial cells, myofibroblasts) undergo apoptosis or move out of the wound, leaving an area rich in the protein component of the extracellular matrix of the skin [30]. Then, collagen I and III fibers are replaced by collagen I fibers (on average 6–12 months after injury).

The result of remodeling with superficial skin trauma is a barely visible scar. In deep trauma, the scar is often seen as a smooth, pale, and flattened scar (normotrophic scar) [3]. Scar tissue is formed by cells (mainly fibroblasts) and disorganized matrix (mainly fibril collagens) [31]. Excessive deposition of extracellular matrix gradually leads to the formation of fibrosis (hypertrophic scar) with loss of skin function. Moreover, the most significant components are transforming growth factor-β (TGF-β), connective tissue growth factor (CTGF), interleukin-4, 13 (IL-4, 13), platelet-derived growth factor (PDGF), and osteopontin [32].

A hypertrophic scar on the skin is an area of fibrous connective tissue consisting of disordered collagen fibers (with an increased content of collagen III in comparison with normotrophic scars), fibroblasts, and increased vascular density [33]. The main risk factors for the formation of hypertrophic scars are gender; age; genetic predisposition; the patient’s immunological reactions; type of injury; the size and depth of the wound; anatomical location of the wound; and mechanical tension of the wound [34].

Another type of scarring is the formation of keloid scars on the skin. Unlike hypertrophic scars, keloid scars go beyond the initial tissue damage (wounds) and do not tend to regress spontaneously. They differ histologically from normal skin and hypertrophic cicatrices in the arrangement of collagen fibers, the presence of α-smooth muscle actin-positive myofibroblasts (α-SMA), and the degree of angiogenesis. In addition, their development is genetically determined [35].

The formation of hypertrophic scars is considered to be the result of an imbalance in the exchange of the extracellular matrix during wound healing [36]. Skin defects in the form of chronic wounds occur when the timely restoration of the structural and functional integrity of the skin has not occurred [37]. This can occur due to persistent inflammation, interruption of keratinocyte migration, and improperly regulated signaling and/or expression of specific microRNAs [38].

Abnormal scarring can be caused by chronic inflammation; excessive production of collagen and various components of the extracellular matrix; violation of the regulation of skin wound healing; dysfunction of enzymes involved in the degradation of damaged skin components; mechanophysiological conditions (for example, excessive tension of the skin in the wound area); and involutional changes in the dermis [39,40].

### 3.2. Extracellular Matrix

The extracellular matrix is a component of all mammalian tissues and provides a bioactive environment that controls the behavior of cells using chemical and mechanical signals [41]. Historically, it was thought to be an inert substance that only serves to support cells. However, with the expansion of data from various basic and clinical studies, it became clear that the components of the extracellular matrix are the main components of the cellular microenvironment and are actively involved in wound healing, due to their ability to influence cell behavior (proliferation, adhesion, and migration). In addition, the extracellular matrix is involved in the regulation of cell differentiation and death through integrins, cytokines, and growth factors [42,43,44]. Thus, the extracellular matrix is essential for the normal development, functioning, and homeostasis of all eukaryotic cells. It actively participates in the regulation of growth factors, receptors, hydration level, and pH of the local tissue environment.

Remodeling of the extracellular matrix occurs under the action of MMPs and growth factors. Remodeling, in turn, is involved in the regulation of cell differentiation processes (maintenance of stem cell niches, angiogenesis, bone remodeling, and wound healing) [42].

Abnormal remodeling of the extracellular matrix can lead to unregulated cell proliferation and invasion, impaired apoptosis and cell differentiation, and impairment of all physiological functions of the skin. The extracellular matrix (in addition to migration, aging, and expression of fibroblast genes) can influence the differentiation of fibroblasts into different cell types. In fact, the fibroblast itself is determined by the local extracellular matrix surrounding it.

With age, the stiffness of the extracellular matrix increases due to an increase in the cross-linking of collagen fibers. With an increase in the rigidity of the extracellular matrix, fibroblasts exhibit a greater expression of α-SMA [45]. Depending on the depth of occurrence, fibroblasts can express different amounts of collagen and mRNA of collagen I and III. Thus, fibroblasts in deeper layers of the dermis produce less collagenase mRNA than fibroblasts in more superficial layers. Fibroblasts located in the papillary dermis are less prone to excessive fibrosis [34].

Fibroblasts are able to change their cell profile. This most common transition is their transition to contractile myofibroblasts, which are alpha smooth muscle actin (α-SMA)-positive cells that are activated by TGF-β1 [46].

In addition, the formation of myofibroblasts from fibroblasts is associated with mechanical stress. Thus, an increase in skin cell elongation by 15% can contribute to a change in focal adhesion kinase signaling or increase the expression of αSMA. This is the basis for the assumptions about the positive effect of botulinum toxin type A on the prognosis of scar formation: due to the paralysis of local muscle fibers, it is possible to reduce the proliferation of myofibroblasts and the production of collagen, reducing the risk of hypertrophic scar formation [47].

All this demonstrates that the balanced activity of fibroblasts is extremely important in the process of skin wound healing. Decreased fibroblast activity can lead to chronic wound formation, and excessive fibroblast activity can lead to excessive fibrosis and keloid scarring.

### 3.3. The Composition of the Extracellular Matrix

The composition of the extracellular substance is heterogeneous and constantly renewed. The following are the main classes of macromolecules identified in the extracellular matrix: glycoproteins (fibronectin, proteoglycans, laminin) and fibrous proteins (collagen, elastin). Proteins related to extracellular matrix (EMC) are called matrisomes. [48]. In addition, the composition of the extracellular matrix includes fibrin; fibronectin; vitronectin; elastin; fibrillin; integrins (receptors associated with the cell membrane); and laminins (secreted molecules that make up part of the basement membrane) [49].

The most abundant protein in the skin is collagen. Collagen accounts for up to 30% of the total protein in the skin. It provides tensile strength to the skin and binds to elastic fibers, which provide tissues with the ability to recover from stretching. The natural form of collagen fiber provides the necessary mobility of the skin during stretching. However, in scar tissue, collagen fibers are straighter and thinner. As a result, the tensile strength of the collagen fiber in the scar tissue decreases. Normally, collagen turnover is slow, but it is accelerated during remodeling and healing of skin wounds [50].

We have already written in detail about collagens in the skin, so in this review we are focusing on the other components of EMC [51]. Mechanobiological interactions between the extracellular matrix and cells are essential for wound healing. This interaction can both control physiological processes and trigger pathological processes. Thus, abnormal compaction of interstitial collagen can lead to pathological fibrosis of the skin after injury [52].

Other extracellular matrix proteins (fibronectin, laminins, and matrix cell proteins) are involved as connectors or binding proteins. Fibrin, fibronectin, and vitronectin are key mediators of hemostasis and cell migration in wound healing.

The most common proteoglycans are hyaluronan, decorin, versican, and dermatopontin. Proteoglycans are composed of large carbohydrates (usually glycosaminoglycans (GAGs)) attached to a protein. Anionic GAGs allow to bind water and other cations (for example, calcium ions). Different types of GAGs can bind to each other. Another function of GAGs is to fill extracellular space and lubricate. Proteoglycans promote cell adhesion to the extracellular matrix. They bind to secreted proteins and growth factors in the extracellular matrix [31]. Thus, versican aggregates with elastic fibers, influencing cell migration, while decorin interacts with collagen and regulates the organization of collagen fibrils [53].

A special type of GAG is hyaluronan (hyaluronic acid), the only GAG that lacks a protein core. Hyaluronic acid plays an important role in a number of biological processes: cell signaling; inflammation; wound healing; cell development; maintenance of tissue homeostasis; and interaction with other elements of the extracellular matrix. Hyaluronic acid plays a role in maintaining tissue hydration and osmotic balance and plays a key role in fibrous and normal wound healing. The action of hyaluronic acid can take opposite forms depending on its molecular weight. Thus, high-molecular-weight hyaluronic acid (more than 500 kDa) reduces inflammation, increases the expression of collagen III, and increases the activity of the anti-fibrotic TGF-β3. Low-molecular-weight (fragmented) hyaluronic acid (less than 400 kDa) increases inflammation, increases the expression of collagen I, increases fibroblast proliferation, and increases myofibroblast migration [31].

Hyaluronic acid acts on fibroblasts through receptors CD44 and RHAMM (receptor for hyaluronan-mediated motility), as well as through autocrine regulation. The large, branched hyaluronic acid molecule traps TGF-β1 molecules near the fibroblast. This promotes the differentiation of fibroblasts into myofibroblasts [54].

The other two GAGs (dermatane sulfate and chondroitine sulfate) can also be found in proteoglycans. Additionally, they can be in a free state in the extracellular matrix. Chondroitin sulfate, dermatane sulfate, and keratan sulfate proteoglycans are structural components of the extracellular matrix of the skin associated with collagen fibrils. They are important for the binding of fibrils to the surrounding extracellular matrix. These GAGs are very similar in structure and differ only in modified sugar (iduronic acid) [31].

The family of small lecithin-rich repetitive proteoglycans (SLRPs) includes decorin, biglycan, fibromodulin, lumican, and asporin. They are involved in the formation of collagen fibrils and the assembly of the extracellular matrix of the skin [55].

Fibronectin is the second most abundant extracellular matrix protein. This protein is a dimeric molecule containing binding domains for many other molecules of the extracellular matrix (for example: collagen, heparin sulfates, integrins). Fibronectins are encoded by a single gene, and their diversity is a consequence of alternative splicing. The final molecule of this protein is formed by dimerization of fibronectin monomers. This process leads to the formation of fibronectin I, II, and III. Fibronectins can be dissolved in the extracellular matrix, or they are associated with other components, anchoring cells to the fibers of the extracellular matrix [56].

Laminins are large cross-shaped interchangeable proteins of the extracellular matrix. They are composed of α, β, and γ-chains and are encoded by 12 genes. Laminin is a trimeric cross-linked glycoprotein commonly found in the basement membrane. Laminin facilitates interactions between skin cells and other components of the extracellular matrix (for example, between heparin sulfate and collagen). Laminins are involved in interaction with cellular receptors and extracellular ligands [57]. Laminin can be a natural inhibitor of TGF-β1, and it can reduce the fibrosis of scars. In addition, laminin binds collagen at the β1-integrin binding sites, which is necessary for normal fibrillogenes. Decreased laminin levels have been found in hypertrophic skin scars following abnormal wound healing [33].

The most common proteoglycan in adult skin is decorin. This protein belongs to the SLRPs family. It has a binding affinity for TGF-β1. In general, decorin regulates the organization of collagen fibers and collagen bundles in the skin, reducing the stimulating effect of TGF-β on the production of collagen, fibronectin, and glycosaminoglycans [55].

Another proteoglycan of the skin is lumican (translucent, predominant in the cornea). It promotes the differentiation and contraction of myofibroblasts through the α2-integrin-mediated pathway and regulates the assembly of collagen fibers in the skin [58]. Additionally, lumican plays an important role in proapoptotic signaling in fibroblasts and may be important for fibrosis after wound healing.

Another proteoglycan from the SLRPs family is dermatopontin. It increases the elasticity of the skin, increases its tensile strength, and enhances the formation of collagen fibers (induces fibrillogenes). In the skin, dermatopontin increases the adhesion of fibroblasts to fibrin fibers of the extracellular matrix and promotes fibrillation of fibrils with a dose-dependent effect [55].

The extracellular matrix is a “reservoir” of signaling molecules, including chemokines; cytokines; and growth factors. These signaling molecules are retained in the extracellular matrix of the skin. For example, TGFβ, coupled with fibrillin and fibronectins, is stored in the extracellular matrix in an inactive form until MMPs activation or mechanical damage to the skin.

Thus, many components (proteins, proteoglycans) and enzymes are involved in the remodeling of the extracellular matrix. By balancing these extracellular matrix components, an optimal wound healing process is achieved.

### 3.4. The Role of Extracellular Matrix in Skin Wound Healing

The dermis contains densely packed collagen fibers that provide the skin with tensile strength. However, when the skin is damaged, a number of processes are launched aimed at preventing the penetration of infection and restoring the integrity of the skin in the damaged area (wound) [59]. Closure of the wound edges occurs along Langer’s lines of tension, which, in turn, are histologically correlated with the orientation of collagen fibers. In the wound, collagen fibers intertwine and create a structural scaffold, allowing cell adhesion, chemotaxis, and migration. Excessive tension on collagen fibers in the early stages of wound healing can lead to the formation of hypertrophic scars. In contrast, a decrease in the tension of collagen fibers with laxity and age-related changes in the skin may be associated with a decrease in the production of collagen I and III at the stage of wound healing.

When a skin wound occurs, enzymes of the extracellular matrix are activated. The most important enzymes in the remodeling of the extracellular matrix are MMPs, disintegrin, and a metalloproteinase from the thrombospondin motif family (ADAMTS).

MMPs are a large family of zinc-dependent endopeptidases involved in the degradation of all major components of the extracellular matrix, including the basement membrane. Initially, MMPs are secreted as inactive zymogens with a propeptide domain that must be removed for MMP activation. MMP precursors include an amino pro-domain masking the catalytic zinc-binding motif [60].

Currently, at least 24 different MMPs are known, which can be soluble and membrane-bound. MMPs are classified according to their structural organization and substrate specificity into: collagenases; gelatinases; stromelysins; matrilisins; and membrane types of MMP. Under physiological conditions, MMP activity is tightly regulated. However, MMP activity increases with pathological processes. Inhibitors such as tissue MMP inhibitors (TIMPs) [61] inactivate MMPs of the extracellular matrix.

The following are involved in the regulation of cell phenotype, adhesion and migration: adamlysins-ADAMs (disintegrin and MMP); ADAMTS (adamlysins with thrombospondin motif) are extracellular matrix proteinases that are involved in the formation of cytokines, the release of growth factors, and degradation of components of the extracellular matrix. Heparanases and sulfatases degrade heparin sulfate, affecting its ability to bind multiple growth factors, altering signaling events [60].

MMPs first destroy collagen I, which restricts the migration of skin stromal cells. Then, MMPs act on elastin fibers, release peptides that act on wound healing, accelerate fibroblast proliferation, and increase collagen I and tropoelastin. These peptides are collectively called matrikines.

Matrikines are biologically active fragments obtained as a result of proteolytic cleavage of collagens, proteoglycans, elastin, and laminins. Thus, hyaluronan fragments regulate inflammation and wound healing. Further, with the interaction of integrin αvβ3 and elastin-binding protein, through protein kinase A, there is improvement in adhesion, migration, and proliferation of fibroblasts. Thus, SLRPs—decorin and lumican—are decoupled and removed from the adjacent matrix [62].

Fibrin, fibronectin, and vitronectin are key mediators of hemostasis and cell migration in wound healing. Fibrin is the first fibrous structure in wounds. It is formed from soluble blood plasma fibrinogen and forms a temporary clot matrix during wound healing. When fibroblasts migrate to the wound area, fibroblasts compress the fibrin matrix and use it as a surface for migration and tissue remodeling, replacing it with collagen and other extracellular matrix proteins [63].

During wound healing, fibronectin is involved in the organization and stabilization of the extracellular matrix. It is required for the deposition of collagen I and other extracellular matrix proteins, and it is also required to regulate the activity of lysyl oxidase, which is involved in strengthening collagen fibers. The plasma fraction of fibronectin is incorporated into the fibrin clot, providing a wound seal and a scaffold for leukocyte and endothelial cell migration. At the proliferation stage, fibronectin assembles into a complex three-dimensional structure on the cell surface, which provides tissue architecture and regulates cell adhesion, migration, proliferation, and apoptosis during skin wound healing. It is believed that the formation of further collagen networking depends on the initial fibronectin structure, through mechanisms involving integrins. Additionally, fibronectin is required for the neovascularization of a healing wound [63].

Stimulation of the proliferative activity of fibroblasts through TGF-β depends on the preliminary assembly of the fibronectin matrix. Fibronectin is commonly present in the acute phases of inflammation and wound remodeling. At low tissue tension, fibronectin binds collagen fibers. Then, a network of fibril collagens is formed, replacing fibronectin fibers and creating a high tension of the extracellular matrix [64].

Insoluble fibronectin bundles are formed from the soluble fraction in blood plasma. In the acute phase of wound healing, fibronectin binds to integrin αvβ3 (expressed by fibroblasts) and stimulates their migration into the wound. Additionally, fibronectin has a site for binding and stabilizing fibrin (a prerequisite for the migration of fibroblasts), and it also interacts with other cells and fibrils involved in wound healing in the skin.

Vitronectin is important for the early contraction of skin wounds. Thus, the creation of tension of collagen fibers in the wound area is provided by fibroblasts, which first attach to fibronectin, then to vitronectin, and only after that to collagen. Vitronectin affects fibroblast proliferation mediated by fibronectin. Vitronectin is a kind of modulator of the migrating and proliferating response of fibroblasts [65].

Another unique component that plays a role in the regeneration of skin wounds is tenascin-C. The expression of this protein in intact tissues is minimal. Expression increases with tissue damage (wound) [38]. Tenascin-C is a matrix and has many repeats of fibronectin-like integrin-binding domains and EGF-like repeats for binding to components of the extracellular matrix and signaling through the EGF receptor. Tenascin-C regulates cell adhesion and thus affects the functionality of the dermis during wound healing. Experimental data on axolotls have shown that low levels of fibronectin and high levels of tenascin-C promote optimal wound healing instead of pathological scarring.

In addition, the role of MMPs in the regulation of fibrotic response has been shown [66]. The greatest increases in osteopontin, tenascin C, TGF-β1, and TIMP1 occur in response to skin damage and an increase in MMP expression in the wound.

During wound healing, pro-migration dermatopontin and anti-migration decorin balance each other and mutually change their activity.

The presence of GAGs is required at the earliest stages of skin wound healing to facilitate the migration of fibroblasts through the CD44 receptors. At the same time, in the fetus (when there is no cicatricle wound healing), GAGs have a large molecule length. In studies comparing the regeneration process in a fetus and an adult wound, the importance of hyaluronic acid has been shown. Therefore, in the fetal wound, when the regeneration ends without scarring, there was a higher content of GAG and higher molecular weight of hyaluronic acid (which reduces angiogenesis and inflammation). The increased content of hyaluronic acid in the skin wound area persisted longer in the fetus than in adults (3 weeks versus 7 days) [44,67,68,69,70].

The secreted glycopeptide fibulin-5 binds and mediates the development of elastic fibers. Under normal conditions, it is inactive, and its expression is activated 14 days after the start of wound healing. Its overexpression induces the formation of granulation tissue and initiates remodeling of the extracellular matrix. At the same time, fibulin-5 does not affect the migration and proliferation of fibroblasts [38].

In addition, the extracellular matrix contains matrix-cell proteins. These proteins are secreted and interact in the extracellular matrix of autocrine and paracrine cells. They do not affect the mechanical structure of the extracellular matrix. Matrix-cellular proteins include osteopontin; osteonectin; thrombospondins −1 and −2; tenacin-C; fibulins; and proteins of the CCN family. These proteins act as signaling molecules that are dynamic over time. They can only be colonized in a skin wound, not present in healthy skin. During wound healing, these proteins act on fibroblasts. In turn, fibroblasts produce more of these proteins in the cutaneous wound. This process is a variant of autocrine regulation [38].

The fairly recently described osteopontin was first discovered in bone. In addition to participating in bone mineralization, osteopontin can also participate in the processes of fibrosis in the skin. It interacts with collagen and fibronectin and also contains several cell adhesion domains that interact with integrins and CD44. Osteopontin increases fibroblast migration and proliferation. It is required for myofibroblast differentiation in response to the TGF-β signal [71].

The glycoprotein most commonly found in bone is osteonectin (a secreted protein that is acidic and rich in cysteine). This protein is also associated with fibrosis in the skin and other tissues. It can increase gene expression and protein assembly, including collagen I.

Another matrix cell peptide, CCN2, is usually not present in the skin but appears when tissue is damaged (skin wounds). It increases the expression of collagen I and III by fibroblasts, tissue inhibitors of MMP, and basic fibroblast growth factor. At the same time, CCN2 does not affect the expression of proteoglycans. In addition, it stimulates the recruitment of mesenchymal stem cells to the wound site for their differentiation into fibroblasts. It is believed that the expression of CCN2 in the skin is associated with the formation of hypertrophic scars [72].

After tissue damage, fibroblasts produce various cytokines and growth factors; they differentiate into a highly contractile phenotype characterized by the expression of α-SMA—myofibroblasts, as described previously [73,74].

### 3.5. Genetic Aspects of the Role of Extracellular Matrix in Wound Healing

Currently, more than 100 genes are known that are responsible for the microenvironment involved in wound healing in the skin [75,76]. Studies in transgenic mice have shown the role of the earliest gene regulators, including the *AP-1 FOS*, and *JUN* genes, as well as zinc finger transcription factors known as Krox, which are involved in the activation of transcription for several hundred other genes that provide cell proliferation [77]. Additionally, the epigenetic regulation is important, as we have already written about in a previous article [51].

Of great interest is the study of single nucleotide polymorphisms (SNPs) of genes responsible for the synthesis of collagen fibers, elastic fibers, and hyaluronic acid in different types of skin wound healing. Mutations in the genes responsible for the synthesis of skin collagen (for examples, collagen I, III, IV, V, VI, VII, XIV, XVI, and XVII types) lead to various skin pathologies, including abnormal wound healing [31].

In studies on mice with collagen III deficiency, spontaneous skin wounds and an uneven diameter of collagen fibrils were noted [78]. With a deficiency of COL3A1 expression in granulation tissue, a higher content of myofibroblasts was noted in experimental animals [79,80,81], and in humans, a mutation in the *COL3A1* gene causes type IV Ehlers–Danlos syndrome, in which skin wounds heal with a large number of scars [44]. Mutations in the *COL1A2* gene lead to an increased risk of hypertrophic scar formation after skin injury [82].

Thus, with Marfan syndrome, caused by mutations in the *FBN1* gene encoding fibrillin-1, there is a decrease in the level of extracellular fibrillin-rich microfibrils, which usually act as a reservoir for TGF-β. As a result, TGF-β signaling is impaired during wound healing [83,84].

A study investigating the role of Lucilia sericata larvae in wound healing showed the highest expression of the *COL1A2*, *COL4A1*, *CTSK*, *CCL7*, *ANGPt1*, *CD40lG*, *EGF*, and *ITGB5* genes in wound healing in an experiment [85]. Another study found a decrease in elastin content during the wound healing [86,87].

At present, studies of SNPs of candidate genes in different types of healing of skin wounds continue.

## 4. Conclusions

Surgical treatment of skin wounds requires an interdisciplinary approach involving doctors of various specialties (dermatologists, plastic surgeons, microsurgeons, traumatologists, etc.). It is important to not only control the infection and ensure rapid closure of the skin wound, but also to be able to manage the healing process of skin wounds [28]. Various methods are proposed to control and improve the healing of wound skin lesions, including invasive (injections of platelet plasma, botulinum toxin type A, glucocorticosteroids) [88] and non-invasive (laser therapy, creams with growth factors and cytokines, silicones) approaches [34,89]. However, no single (“gold”) standard for managing skin wound healing has been developed [90].

In recent decades, bioengineering techniques have been developed that temporarily replace a skin defect [91], along with targeted drugs that block a specific signaling pathway for skin wound healing. Thus, blockading the TGFβ signaling pathway is considered a promising method [58,92,93].

This review shows the significant role of the extracellular matrix of the skin in wound healing. Understanding the role of the extracellular matrix of the skin is important when we plan scar treatment. Before surgery, it is important to assess the risk of abnormal wound healing. Therefore, such risk assessment also includes consideration of violations of the functions of the extracellular matrix of the skin. 

The search for biomarkers (predictors, risk factors) of pathological scarring of skin wounds continues. Along with external damaging factors and hereditary diseases, multifactorial pathology is of undoubted interest, including the carriage of risk alleles for SNP of candidate genes associated with abnormal wound healing. Therefore, additional fundamental and clinical studies are needed to study the mechanisms of pathological scarring and the possibilities of combination therapy to prevent pathological skin wound healing.

## Data Availability

Not applicable.
